# Development of Polypropylene-Based Single-Polymer Composites With Blends of Amorphous Poly-Alpha-Olefin and Random Polypropylene Copolymer

**DOI:** 10.3390/polym12061429

**Published:** 2020-06-26

**Authors:** László József Varga, Tamás Bárány

**Affiliations:** Department of Polymer Engineering, Faculty of Mechanical Engineering, Budapest University of Technology and Economics, Műegyetem rkp. 3., H-1111 Budapest, Hungary; vargalj@pt.bme.hu

**Keywords:** single-polymer composites, polypropylene, amorphous poly-alpha olefin, impact test

## Abstract

We developed polypropylene-based single-polymer composites (PP-SPC) with blends of amorphous poly-alpha-olefin (APAO) and random polypropylene copolymer (rPP) as matrix material and polypropylene (PP) woven fabric as reinforcement. Our goal was to utilize the lower melting temperature of APAO/rPP blends to increase the consolidation of the composites and decrease the heat load of the PP reinforcement. We produced the composites by film-stacking at 160 °C, and characterized the composites with density, peel, static tensile and dynamic falling weight impact tests, and by scanning electron microscopy. The results indicate that consolidation can be enhanced by increasing the APAO content of the matrix. We found that the APAO content of 50% is optimal for tensile properties. With increasing APAO content, the perforation energy decreased, but even the well-consolidated composites showed very high perforation energy. In the case of a pure APAO matrix, fiber content can be increased up to 80 wt% without a severe loss of consolidation, resulting in good tensile properties. The PP-SPCs developed possessed excellent mechanical properties, and well-consolidated composites can be produced with APAO/rPP blends as a matrix with high fiber content.

## 1. Introduction

Nowadays, polypropylene (PP) is one of the most widely used polymers in many applications in everyday life, because of its relatively good mechanical properties and low price [1]. However, to satisfy the demands of industrial applications, the properties of PP are enhanced, mostly with glass fiber (GF) reinforcement [2]. Nowadays, as the amount of plastic waste is formidably high, and public opinion has shifted towards environmental consciousness, recycling has become of paramount importance in the plastic industry [3,4,5]. The main problem with the recycling of glass fiber-reinforced PP is that its mechanical properties strongly deteriorate during recycling due to fiber breakage [6]. Although it is possible to extract polypropylene from composites with the use of certain solvents, these solvents are usually hazardous materials [7]. Another significant drawback of PP/GF composites is that fiber/matrix adhesion is often unsatisfactory [8].

Single-polymer composites (SPC), where the matrix and the reinforcement belong to the same material family, do not have this problem. The concept of one-polymer composites, in which the components are of the same polymer, was first described by Capiati and Porter in 1975 [9]. Since then, several methods have been developed to produce single-polymer composites. One of these techniques is hot compaction, initially invented by Hine and Ward [10]. Hot compaction is based on the selective melting of the surface of the reinforcing fibers under suitable conditions of pressure and temperature, exploiting the fact that the surface of the fibers has a different melting behavior from their core. The melted surface of the fibers subsequently becomes the matrix material in the composites after solidifying. Hot compacted composites have extremely good fiber/matrix adhesion because their matrix and reinforcement have the same chemical composition (i.e., derived from the same material). However, due to the tight processing window, the processing temperature needs to be chosen and maintained very carefully to avoid excessive fiber melting [10,11,12].

To widen the processing window, a copolymer can be used as the matrix material. Peijs et al. consolidated coextruded tapes at a temperature over the melting temperature of the matrix, and under pressure and created PP-based single-polymer composites. In this technique, the outer layer to be melted is a copolymer and acts as the matrix [13,14,15,16,17].

Film stacking is also an important SPC production method. In the film stacking process, layers of polymer sheets and reinforcing fabric are placed on top of each other, and compression molded under suitable conditions of pressure and temperature. At the elevated temperature, the polymer sheets melt, impregnate the reinforcing fabric, and become the matrix after cooling. Single-polymer composites are often described with their quality of consolidation. Good consolidation mainly refers to a minimal void content between the fiber and the matrix [18]. Since in film stacking, the matrix needs to impregnate the reinforcement (originally, there is no matrix between the fibers), it is crucial to provide a proper difference between the melting temperatures of the matrix and the reinforcement to ensure a good level of consolidation. In the case of polypropylene, one way to provide this difference is to use PP homopolymer as reinforcement, and a PP copolymer (most commonly random polypropylene copolymer—rPP) as matrix [19,20,21]. Another possibility is to utilize the polymorphism of polypropylene, as the β crystalline form of PP has a lower melting temperature than the α-form. The β-form can be produced by using selective β–nucleating agents as additives [22]. The mechanical properties [23,24,25,26,27,28], failure behavior [29,30], and reprocessability [31] of these PP-based composites of different crystalline forms have been studied extensively.

Although polypropylene is proved to be a viable material to form single-polymer composites due to its relatively good mechanical properties and low price, the above-mentioned SPC production methods have been successfully applied to other polymers as well. Polyethylene terephthalate (PET) is a promising candidate to form SPCs due to its high melting temperature, consequently, the high-temperature applications of PET-based SPCs are less limited. Furthermore, the processing temperature window can be widened with the combination of PET homopolymer reinforcement and a copolymer matrix [32,33]. Biodegradable polymers, such as poly(lactic acid) (PLA), also can be ideal materials to form SPCs, as they can help to further decrease the ecological footprint of these composites [34,35].

In the present study, we suggest a new approach of using blends of amorphous poly-alpha-olefin (APAO) and random polypropylene copolymer as matrix to widen the processing window of the SPCs. APAOs were invented for melt adhesive applications to replace atactic polypropylene (aPP), which is a by-product of isotactic polypropylene (iPP) production. As opposed to aPP, the properties of APAOs can be tailored to satisfy the demands of different applications. Although APAOs are often copolymers containing ethylene or 1-butene, they mostly consist of propylene repeating units arranged in an atactic manner [36]. Nam et al., reported that increasing aPP content significantly improves the impact properties of aPP/iPP blends [37]. As APAOs are similar to aPP, this could further improve the energy damping ability of APAO-based SPCs. Furthermore, the “stickiness” of APAO (nowadays it is mainly used in melt adhesives) can be reduced if rPP is added. We produced polypropylene-based single-polymer composites by film stacking with different APAO/rPP blends as matrix.

## 2. Materials and Methods

### 2.1. Materials

**Figure 1:**
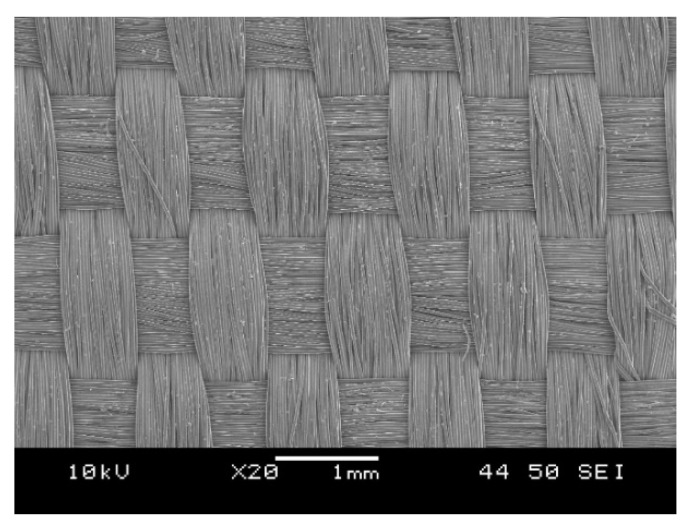
Scanning electron microscopic image of the reinforcing woven fabric.

We selected a woven fabric composed of high-strength polypropylene multifilament (Lanex a.s., Bolatice, Czech Republic) with a nominal weight of 178 g/m^2^. The multifilament has a linear density of 550 dtex, a tenacity of 6.7 cN/tex and an elongation at break of 20%. A single reinforcing fiber has a melting temperature of 171.6 °C (determined by DSC). Its average diameter is 27.6 ± 0.6 µm (measured on 20 single fibers); tensile strength is 558 ± 26 MPa; tensile modulus is 6282 ± 578 MPa; elongation at break is 28.7 ± 2.7%. A roving contains 100 single polypropylene fibers. The woven fabric (Figure 1) was prepared by Csendes and Csendes Ltd. (Szigetbecse, Hungary) upon our request. The properties of the fabric: warp/weft ratio: 53/47%; areal density: 178 g/m^2^; breaking strength (strip) in warp and weft direction: 49.4 ± 0.4 and 44.0 ± 0.6 N/mm, respectively.

As matrix materials, we used blends of R1059A random polypropylene copolymer (MOL Petrolkémia Zrt., Tiszaújváros, Hungary) and a propene-rich amorphous poly-alpha-olefin-based melt adhesive (VESTOPLAST^®^ 792, kindly provided by Evonik Resource Efficiency GmbH, Marl, Germany). The random polypropylene copolymer used has a melt flow index (230 °C, 2.16 kg) of 84 g/10 min, and tensile strength, and modulus of 29 MPa and 1000 MPa, respectively. The APAO used has a molecular weight of M_n_=23,800 g/mol and M_w_=118,000 g/mol, and the melt viscosity of 120 Pas (at 190 °C).

### 2.2. Blending and Extrusion

The blends were produced with a LE 25–30/C corotating twin-screw extruder (Labtech Engineering Co. Ltd., Samutprakarn, Thailand) at 185 °C; the rPP/APAO ratios were 100/0, 75/25, 50/50, 25/75 and 0/100. These matrix materials are referred to as rPP, APAO-25, APAO-50, APAO-75, and APAO, respectively.

We extruded thin sheets from the blends with an LCR300 flat film line (Labtech Engineering Co. Ltd., Samutprakarn, Thailand). The temperature of the die was 185 °C in the case of rPP, APAO-25, APAO-50, and APAO-75. In the case of pure APAO, the steady production of the film could only be ensured at 120 °C due to the low viscosity and extreme “stickiness” of the APAO. Furthermore, as APAO content was increased, the blends showed increasingly similar properties to those of APAO. Because of this phenomenon, we were able to produce only thicker films with higher amounts of APAO.

### 2.3. Composite Preparation

**Figure 2:**
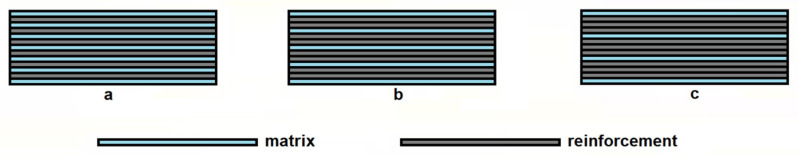
The arrangement of the composites: (**a**) rPP, APAO-25, APAO-50, APAO-75, APAO, (**b**) APAO-2F, (**c**) APAO-3F.

The composites were produced by film stacking at 160 °C, with a Polystat 300S hydraulic press (Maschienenfabric Fr. Schwabenthan & Co. Kg., Berlin, Germany). First, we inserted the laminates into the preheated mold and kept them at 160 °C for 30 s without pressure, then the pressure was increased to 5 MPa, which was maintained for 90 s. Finally, the composites were cooled down to ambient temperature under pressure. The composites were composed of 6 layers of reinforcing fabric, and 7 layers of matrix film (Figure 2a). The composites are referred to according to the name of the matrix they contain (rPP, APAO-25, APAO-50, APAO-75, and APAO). Because of the increased thickness of the matrix films, the fiber content of the composites decreased with increasing APAO content of the matrix. To make the effects of the APAO content of the matrix on the properties of the composites comparable, we normalized the tensile properties and perforation energy to 60 wt% fiber content.

On the other hand, the extremely low viscosity of APAO allowed us to increase the fiber content of the composites by changing the arrangement of the film-stacked package (because of the above-mentioned properties of APAO, producing thinner APAO foils in our apparatus was not possible). The altered arrangements consisted of 5 layers of matrix foil and 8 layers of fabric (one layer of matrix foil after every second fabric layer, Figure 2b), and 4 layers of matrix foil and 9 layers of fabric (one layer of matrix foil after every third fabric layer, Figure 2c). These composites are referred to as APAO-2F and APAO-3F, respectively. In this case—as the goal is to investigate the effects of fiber content—we did not normalize the properties of the composites.

### 2.4. Characterization Methods

The melt flow index (MFI) values of the matrix blends were measured with a CEAST 7027.000 Melt Flow Tester (Instron, Norwood, MA, USA) under 2.16 kg at 160 °C. In the case of APAO, the MFI values were also determined at 120 and 140 °C with the same weight.

Differential Scanning Calorimetry was performed on the blends with a Q2000 DSC device (TA Instruments, New Castle, DE, USA) in a 50 mL/min nitrogen atmosphere, with a heating rate of 10 °C/min.

The shear modulus of the blends was measured with a DMA Q800 device (TA Instruments, New Castle, DE, USA) in an air atmosphere. We conducted the test according to the EN ISO 6721-2 standard on 10 × 10 × 2 mm rectangular specimens with the frequency of 1 Hz with a shear sandwich clamp. The test was conducted from −60 °C to 90 °C with the heating rate of 3 °C/min, and the shear modulus was determined at 23 °C.

We characterized the matrix films and the composites with static tensile tests. In the case of the matrix films, the tests were performed with a Z005 tensile tester (Zwick GmbH & Co., Ulm, Germany) with a crosshead speed of 100 mm/min. The Type 5 tensile specimens were cut out from the matrix films according to the ISO 527-3 standard. In the case of the composites, the test was performed on 25 × 200 mm specimens with a Z250 tensile testing machine (Zwick GmbH & Co., Ulm, Germany) with a crosshead speed of 5 mm/s.

The matrix blends and the composites were investigated with instrumented falling weight impact (IFWI) tests, with a CEAST 9350 falling weight impact testing machine (Instron, Norwood, MA, USA). In the case of the matrix blends, we used 80 mm × 80 mm × 2 mm injection molded (Arburg Allrounder, 420C, Arburg GmbH, Lossburg, Germany) specimens. These specimens were prepared with the injection pressure of 500 bar and the packing pressure of 300 bar. The temperature of the mold was 50 °C, and the melt temperature was 180 °C in the case of rPP, APAO-25, APAO-50, and APAO-75, and 120 °C in the case of APAO. In the case of the composites, the tests were performed on 100 × 100 mm square specimens. In both cases, we conducted the IFWI tests with the following settings: the total mass of the dart was 28.41 kg, falling height was 1 m, impact energy was 278.65 J, dart diameter (semispherical) was 20 mm, and the diameter of the supporting ring was 40 mm. In the case of composites with the APAO-100 matrix, we also performed the test at −30 °C.

Scanning electron microscopic (JSM 6380LA, Jeol Ltd., Tokyo, Japan) images were taken of the microtomed cross-section of the composites with the accelerating voltage of 15 kV and the spotsize of 40. Before the SEM investigation, the specimens were sputter-coated with gold in an argon atmosphere.

The density of the composites was determined according to Archimedes’ law on 10 × 10 mm square specimens (volume was measured in ethanol).

The peel strength was determined with a peel test on 25 mm × 300 mm rectangular specimens with a Z250 tensile testing machine (Zwick GmbH & Co., Ulm, Germany) equipped with a 20 kN force load cell, with a preload of 1 N, and a crosshead speed of 152 mm/min. In the case of APAO-3F, the last two reinforcing layers and one matrix layer were peeled off, while in the case of the other composites, the last matrix layer and one reinforcing layer were peeled off during the test. To initiate peeling, we inserted a thin polytetrafluoroethylene (PTFE) foil between the appropriate layers during lamination.

The tests were performed at room temperature, where it is not indicated otherwise. At least five specimens were tested in all cases.

## 3. Results and Discussion

### 3.1. Characterization of the Raw Materials

**Figure 3:**
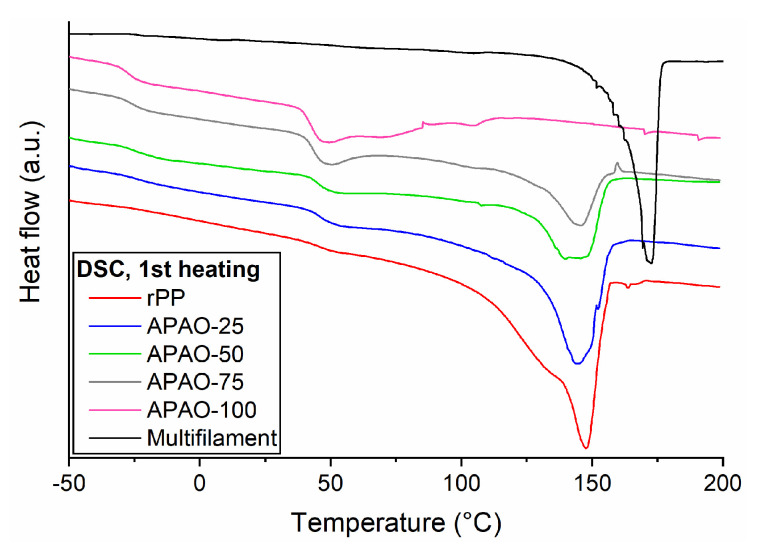
1st DSC heating curves of the rPP/APAO blends.

Although increasing APAO content did not lower the melting temperature of the matrices significantly, it considerably increased the MFI values (Table 1). Nevertheless, the melting temperatures of rPP, APAO-25, APAO-50, and APAO-75 are approximately 20 °C lower than that of the reinforcing fabric, which provides an acceptable processing window (Figure 3). On the other hand, the melting temperature of APAO is far below the melting temperature of the other matrices tested.

During the production of SPCs, it is crucial to avoid the excess heat load of the fabric to prevent relaxation. Although the low melting temperature of APAO would provide a very wide processing window (it showed an MFI value of 30.7 ± 1.7 g/10 min, and 107.3 ± 4.1 g/10 min at 120 °C and 140 °C, respectively), the goal of this study is to investigate the applicability of the rPP/APAO blends as matrix material. To make it possible to compare these matrices with widely different MFI values, we prepared all the single-polymer composites at the consolidation temperature of 160 °C.

**Figure 4:**
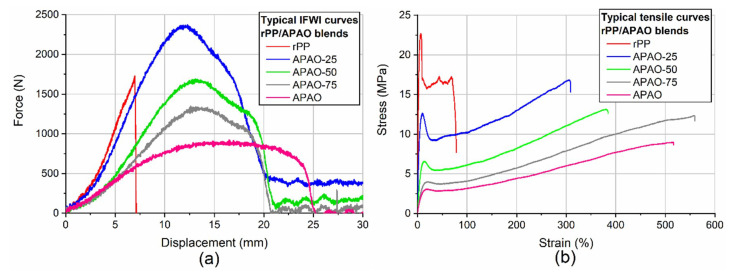
Typical (**a**) IFWI and (**b**) tensile curves of the rPP/APAO blends.

During the IFWI test, rPP had the lowest perforation energy, and it showed brittle failure (Figure 4a). The other blends displayed more ductile behavior, but with increasing APAO-content, the maximal force decreased. Rising of the APAO content considerably reduced the yield stress and tensile modulus, and increased the failure strain of the matrices (Figure 4b, Table 2).

### 3.2. Microstructure of the Composites

With increasing APAO content of the matrix, density increased (Table 3). This result indicates that consolidation was better in the case of the composites with matrices containing more APAO. On the other hand, in the case of lower APAO content, the deviation values were relatively high. This could be caused by the poorer consolidation of the composites.

**Figure 5:**
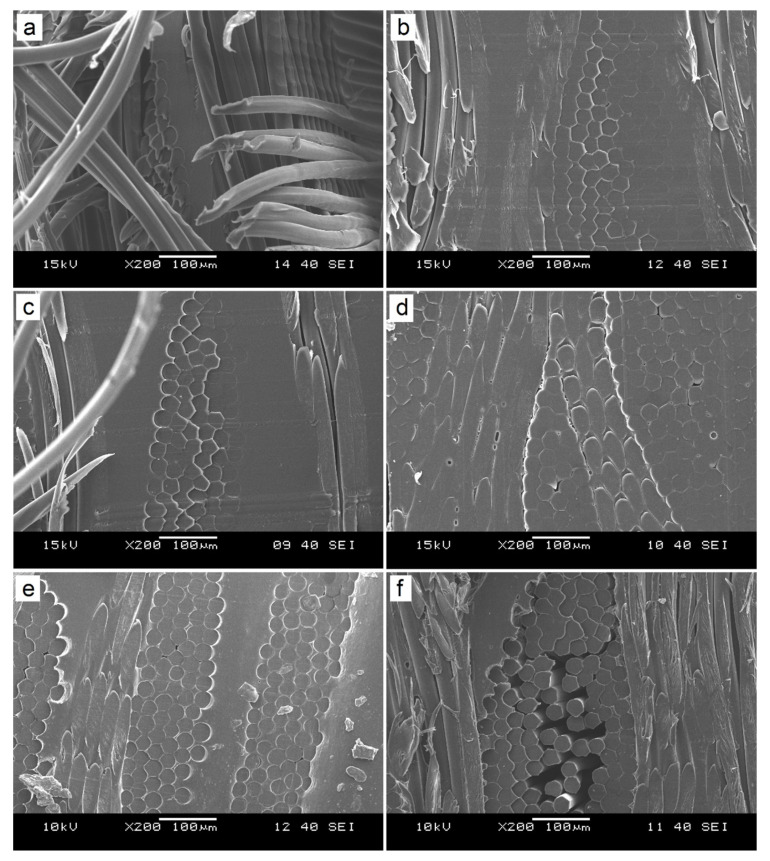
Scanning electron microscopic images of (**a**) rPP, (**b**) APAO-50, (**c**) APAO-75, (**d**) APAO, (**e**) APAO-2F and (**f**) APAO-3F.

In the case of APAO-2F, only a small decrement is detectable in the density, indicating that the APAO matrix can impregnate two layers of reinforcing fabric without significantly impairing consolidation. However, the APAO-3F composite showed remarkably lower density, possibly because the matrix could not impregnate three fabric layers properly, which resulted in poorer consolidation. In the case of rPP, separated matrix and fabric layers can be seen (Figure 5a). The matrix did not impregnate the reinforcing fabric properly, which indicates poor consolidation. Furthermore, because of the poor consolidation, the composite with the rPP matrix could not be appropriately microtomed. As the APAO content of the matrix was increased, consolidation slightly improved, but the composites kept their original laminate-like structure, even at 75% APAO content of the matrix (Figure 5c). In the case of APAO (Figure 5d), only a few voids can be detected; there cannot be seen any separated matrix layers, as the matrix is between the fibers of the reinforcing fabric. Consequently, the matrix properly impregnated the fabric, which indicates good consolidation.

It is also observable in the SEM pictures (Figure 5) that in the case of the composites with low APAO content (up to 75%), the shape of the fibers was deformed, and the originally round fibers became hexagon-shaped due to the pressure applied at the temperature set (160 °C which is close to the melting temperature of the reinforcement). The fibers residing along with the matrix layer, however, kept their original shape. Furthermore, this deformation phenomenon is less observable in the case of the composite prepared with pure APAO matrix. The possible reason for this behavior is that the matrices with low APAO content (hence with larger viscosity) were not able to properly impregnate the fabric layers and to fill the spaces between the fibers. Hence, the softened fibers had to fill those spaces by deformation when the pressure was applied. On the other hand, the APAO matrix was able to impregnate the fabric and to occupy the spaces between the fibers. Consequently, the deformation of the fibers was more constrained, which resulted in a more intact fiber geometry.

**Figure 6:**
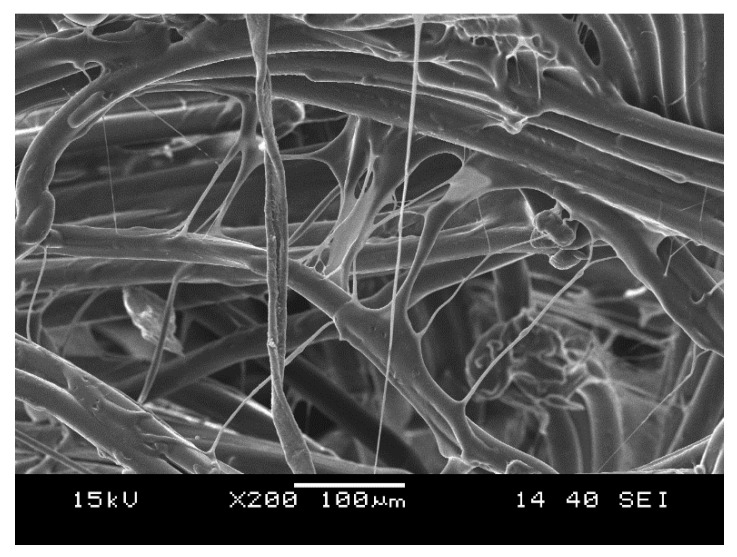
Scanning electron microscopic picture of the IFWI failure surface of the APAO-3F composite

It can be seen in the SEM pictures taken of the failure surface of the APAO-3F composite (Figure 6) that the matrix coats the surface of the fibers and that the matrix suffered severe deformation during the IFWI test. This clearly shows the immense failure strain of APAO, and also proves excellent adhesion between the polypropylene fibers and the APAO matrix.

The cross-section of APAO-2F (Figure 5e) is similar to that of APAO, as the matrix could properly impregnate two layers of fabric. On the other hand, in the case of APAO-3F, several voids can be detected. The fiber contents of APAO-2F and APAO-3F were 77.5 and 82.9%, respectively, so the fiber content of approximately 80 wt% can be achieved with this composite preparation method without significantly reducing consolidation.

With increasing APAO content of the matrix, peel strength strongly increased, which indicates better consolidation. Furthermore with increasing APAO content the stickiness of the matrix blends is also increased, so the fiber/matrix adhesion improved as well. Paralell to this effect the mechanical properties (shear modulus) of the matrix dropped, hence the achievable peel strength is lower than for example for rPP/PP single polymer composites consolidated at higher temperature (3 N/mm [27]). It is also observable that in the case of the APAO matrix, increasing fiber content reduced the peel strength because after the matrix impregnated the increased amount of fabric, there did not remain enough matrix between the reinforcing fabric layers to form a well-functioning bonding layer.

### 3.3. Static Tensile Properties of SPCs

**Figure 7:**
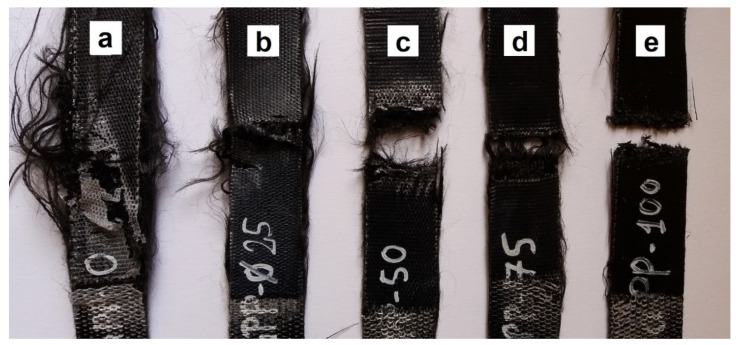
Typical failure behavior of (**a**) rPP, (**b**) APAO-25, (**c**) APAO-50, (**d**) APAO-75 and (**e**) APAO.

**Figure 8:**
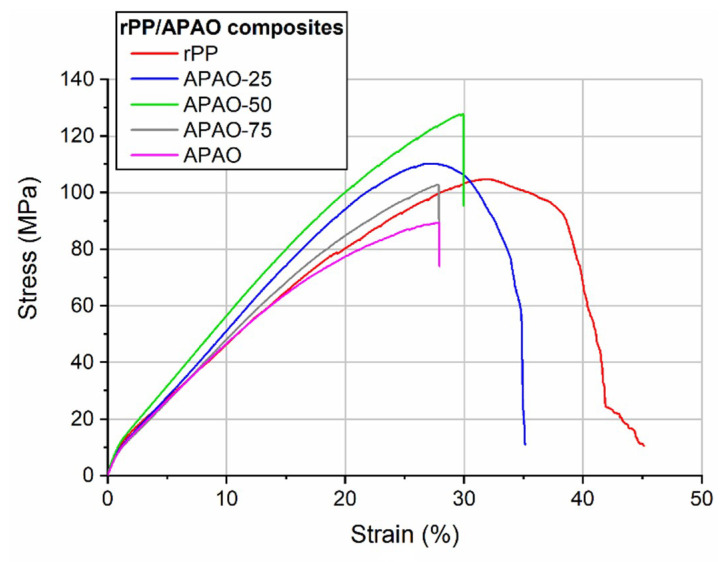
Typical tensile curves of the rPP/APAO composites.

The typical failure behavior of the rPP composite was delamination (Figure 7). With increasing APAO content, fiber breakage became the typical failure behavior. This can also be noticed on the typical tensile curves (Figure 8), as in the case of APAO-50, APAO-75, and APAO, the composites suffered an abrupt failure, indicating fiber breakage.

**Figure 9:**
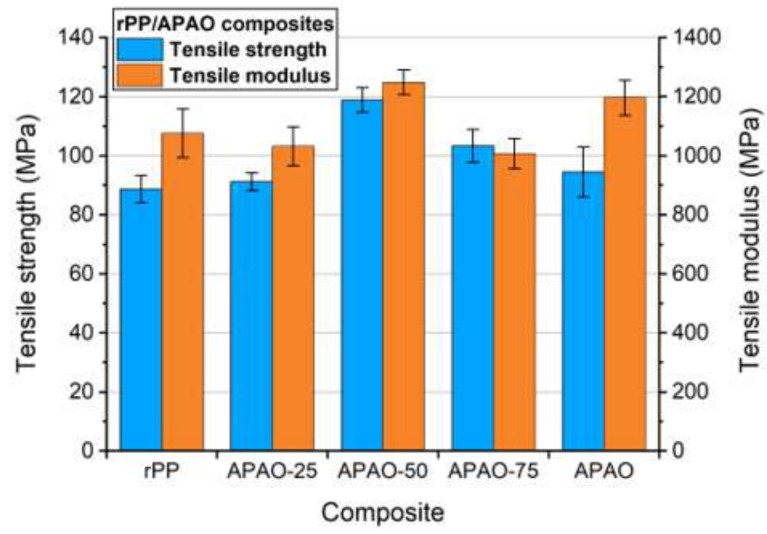
Maximal strength and tensile modulus values of the composites

With increasing APAO content of the matrix, the maximal strength increased up to 50% APAO, above which it decreased (Figure 9). The possible reason for this behavior is that with increasing APAO content, the consolidation of the composites became better, which improved the tensile properties of the composite. On the other hand, the increasing APAO content harshly deteriorated the tensile properties of the matrix (Table 2). Consequently, at higher APAO contents, the matrix was not able to convey the load to the reinforcement properly, so the composite lost its initial integrity during the test. These phenomena affected the tensile properties of the composites in the opposite direction, and the optimal balance between these effects was around 50% APAO content.

The tensile modulus increased with improving consolidation (Figure 9), except for APAO-75, which showed lower modulus. Although the reason for this behavior of APAO-75 is not clear yet, it may be due to the lower density (i.e., poorer consolidation) of APAO-75.

**Figure 10:**
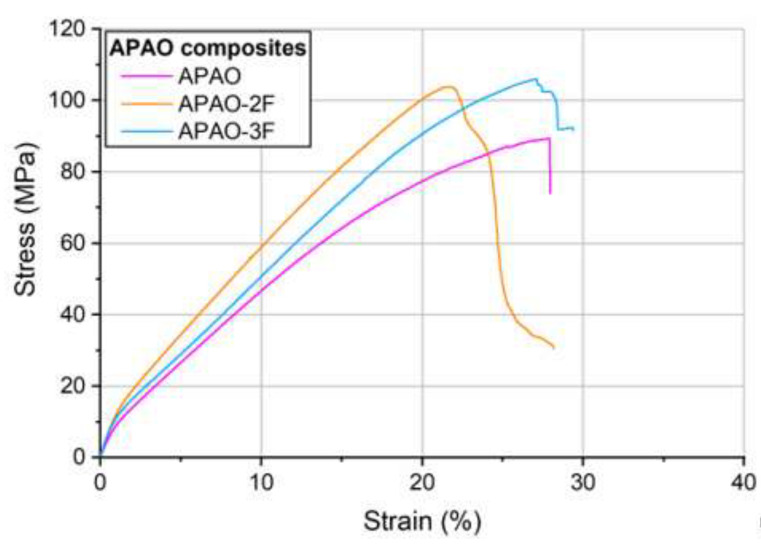
Typical tensile curves of the composites with an APAO matrix with different arrangements.

Increasing fiber content considerably increased maximal strength up to the fiber content of 77.5% (Figure 10, Table 3). Increasing the fiber content to 82.9% did not result in a significant further increment in maximal strength, possibly because of the deteriorating effect of the reduced consolidation. The tensile modulus was not affected by fiber content.

### 3.4. IFWI Tests of SPCs

**Figure 11:**
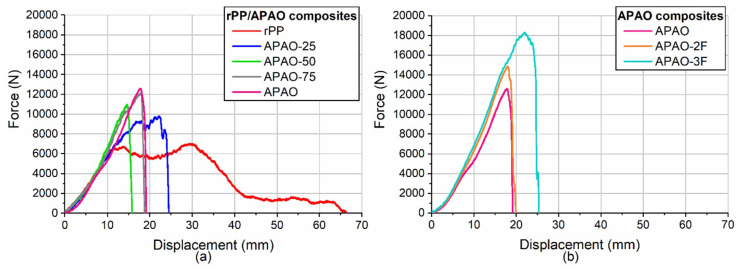
(**a**) typical IFWI curves of the composites with rPP/APAO blends as matrices and (**b**) the typical curves of the composites with increased fiber content.

**Figure 12:**
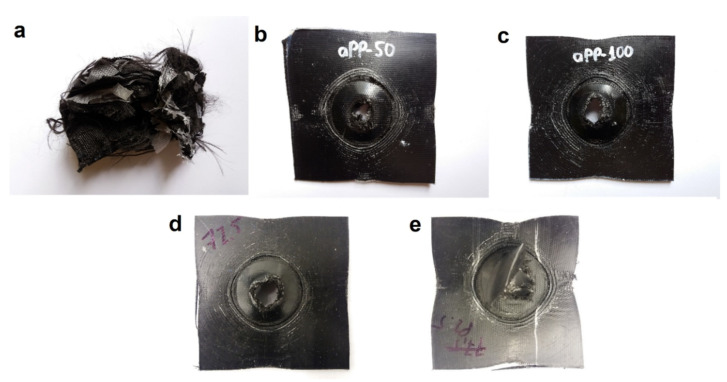
Typical impact failure behavior of (**a**) rPP, (**b**) APAO-50, (**c**) APAO, (**d**) APAO-2F and (**e**) APAO-3F.

The testing of rPP composites was not possible because they underwent severe delamination and also, the machine was not able to clamp the composite sheet properly. This behavior of rPP can clearly be noticed on the typical IFWI curves of the composites (Figure 11). The severe delamination of the rPP composite can also be noticed in the typical failure behavior (Figure 12a). The delamination was caused by poorer consolidation. The other composites, including the ones with the altered arrangement, suffered an abrupt failure, indicating that the clamping unit was able to maintain the position of the specimens upon impact. With increasing APAO content of the matrix, delamination was less observable because of the better consolidation. Increasing the fiber content of the APAO matrix to 77.5% did not result in a significant change of the typical failure behavior (Figure 12d), but a further increase fiber content to 82.9% caused delamination due to poorer consolidation (Figure 12e).

**Figure 13:**
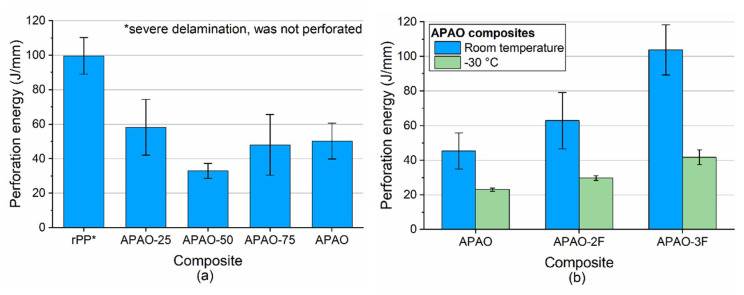
(**a**) perforation energy of the composites and (**b**) perforation energy of the composites with different reinforcement content

The perforation energy decreased with increasing APAO content of the matrix up to the APAO content of 50% (Figure 13a), due to improving consolidation. This can be attributed to the increased energy damping capability of delamination occurring at lower APAO contents. APAO-75 and APAO showed higher perforation energy compared to APAO-50. Although the reason for this behavior is not clear yet, it could be attributed to the decreasing shear modulus of the matrices with increasing APAO content (Table 2), as considerable shear is induced by the dart between the fabric layers upon impact.

In the case of APAO (Figure 13b), increasing fiber content resulted in higher perforation energy values. This behavior was caused by increased delamination due to the poorer consolidation at higher fiber contents, which caused larger displacements.

It is a similar tendency at −30 °C. In this case, the overall perforation energy values are lower because the test temperature of −30 °C is below the glass transition temperature (T_g_) of both the matrix and the reinforcement. The shear modulus of the matrix at room temperature is 5.0 MPa, which was increased to 21.4 MPa at −30 °C. This increment caused brittle behavior.

## 4. Conclusions

In this study, we developed polypropylene-based single-polymer composites with the blends of amorphous poly-alpha-olefin (APAO) and random polypropylene (rPP) as matrix material, utilizing the lower melting temperature of APAO/rPP blends to increase the consolidation of the composites. A woven fabric composed of high-strength polypropylene multifilament was used as reinforcement. We produced the composites by film stacking at 160 °C.

Although the increasing APAO content harshly deteriorated the tensile properties of the matrix, these properties could be strongly enhanced by the incorporation of the reinforcing fabric. Increasing APAO content of the matrix increased the melt flow index and slightly decreased the melting temperature, even though the MFI of pure APAO is much higher than that of the APAO/rPP blends. Consequently, the consolidation of the composites can be improved by increasing the APAO content of the matrix. Based on the tensile tests, the APAO content of 50% seems to be optimal for tensile properties. With increasing APAO content, perforation energy decreased because of the reduced delamination due to the improving consolidation, but even the well-consolidated composites showed very high perforation energy.

The pure APAO matrix showed extremely low viscosity; it could impregnate more layers of reinforcing fabric without a serious loss of consolidation. High fiber content (up to 80 wt%) is achievable with a pure APAO matrix, strongly increasing the tensile properties of the composites.

Although the APAO-based single polymer composites showed several beneficial properties, APAO is an extremely “sticky” material, consequently, it is very hard to use conventional production methods with APAO. A new processing method needs to be developed to ensure steady SPC production with controllable fiber content.

## Figures and Tables

**Table 1:** Thermal properties and MFI values of the matrix blends.

Matrix Blend	Glass Transition Temperature, T_g_ (°C)	Melting Temperature, T_m_ (°C)	Density (g/cm^3^)	Melt Flow Index, 160 °C, 2.16 kg (g/10 min)
rPP	−24.9	147.9	0.918	13.0 ± 0.4
APAO-25	−23.3	145.5	0.911	20.7 ± 0.3
APAO-50	−25.6	144.9	0.903	34.6 ± 1.2
APAO-75	−26.8	144.5	0.892	46.9 ± 0.4
APAO	−27.3	73.9	0.870	163.0 ± 14.8

**Table 2:** Mechanical properties of the matrix blends and their films.

Matrix Blend	Shear Modulus at 23 °C (MPa)	Thickness ^1^ (µm)	Yield Strength ^1^ (MPa)	Young’s Modulus ^1^ (MPa)	Strain at Break ^1^ (%)	Perforation Energy (J/mm)
rPP	25.7	72	23.5 ± 2.7	1032 ± 225	67 ± 6	2.4 ± 0.1
APAO-25	22.7	76	13.5 ± 4.5	350 ± 88	309 ± 4	13.8 ± 0.2
APAO-50	18.5	104	7.4 ± 1.2	124 ± 22	393 ± 30	10.2 ± 0.1
APAO-75	14.7	124	3.8 ± 0.2	58 ± 7	517 ± 62	8.1 ± 0.1
APAO	5.0	133	1.5 ± 0.2	13 ± 3	378 ± 8	8.1 ± 0.1

^1^ Measured on films.

**Table 3:** Fiber content, density, peel strength and tensile properties of the composites.

Composite	Fiber Content (%)	Density (g/cm^3^)	Peel Strength (N/mm)	Maximal Strength (MPa)	Tensile Modulus (MPa)
rPP	70.4	0.874 ± 0.011	0.51 ± 0.11	88.7 ± 4.6	1076 ± 82
APAO-25	69.2	0.876 ± 0.006	0.89 ± 0.15	91.2 ± 3.0	1032 ± 66
APAO-50	62.2	0.883 ± 0.004	1.18 ± 0.16	118.9 ± 4.2	1248 ± 42
APAO-75	58.0	0.877 ± 0.003	1.28 ± 0.06	103.4 ± 5.6	1007 ± 51
APAO	56.2	0.883 ± 0.001	1.98 ± 0.19	94.6 ± 8.5	1196 ± 60
APAO-2F	77.5	0.880 ± 0.002	1.52 ± 0.27	99.7 ± 9.2	1380 ± 66
APAO-3F	82.9	0.869 ± 0.003	1.34 ± 0.18	103.6 ± 4.8	1377 ± 73
